# Interpretable deep learning reveals the role of an E-box motif in suppressing somatic hypermutation of AGCT motifs within human immunoglobulin variable regions

**DOI:** 10.3389/fimmu.2024.1407470

**Published:** 2024-05-28

**Authors:** Abhik Tambe, Thomas MacCarthy, Rushad Pavri

**Affiliations:** ^1^ Department of Biochemistry and Cell Biology, Stony Brook University, Stony Brook, NY, United States; ^2^ Department of Applied Mathematics and Statistics, Stony Brook University, Stony Brook, NY, United States; ^3^ Research Institute of Molecular Pathology (IMP), Vienna, Austria; ^4^ Peter Gorer Department of Immunobiology, School of Immunology & Microbial Sciences, King’s College London, London, United Kingdom

**Keywords:** somatic hypermutation (SHM), activation induced deaminase (AID), immunoglobulin heavy chain, deep learning, integrated gradients, E-box transcription factors, E2A

## Abstract

**Introduction:**

Somatic hypermutation (SHM) of immunoglobulin variable (V) regions by activation induced deaminase (AID) is essential for robust, long-term humoral immunity against pathogen and vaccine antigens. AID mutates cytosines preferentially within WRCH motifs (where W=A or T, R=A or G and H=A, C or T). However, it has been consistently observed that the mutability of WRCH motifs varies substantially, with large variations in mutation frequency even between multiple occurrences of the same motif within a single V region. This has led to the notion that the immediate sequence context of WRCH motifs contributes to mutability. Recent studies have highlighted the potential role of local DNA sequence features in promoting mutagenesis of AGCT, a commonly mutated WRCH motif. Intriguingly, AGCT motifs closer to 5’ ends of V regions, within the framework 1 (FW1) sub-region1, mutate less frequently, suggesting an SHM-suppressing sequence context.

**Methods:**

Here, we systematically examined the basis of AGCT positional biases in human SHM datasets with DeepSHM, a machine-learning model designed to predict SHM patterns. This was combined with integrated gradients, an interpretability method, to interrogate the basis of DeepSHM predictions.

**Results:**

DeepSHM predicted the observed positional differences in mutation frequencies at AGCT motifs with high accuracy. For the conserved, lowly mutating AGCT motifs in FW1, integrated gradients predicted a large negative contribution of 5’C and 3’G flanking residues, suggesting that a CAGCTG context in this location was suppressive for SHM. CAGCTG is the recognition motif for E-box transcription factors, including E2A, which has been implicated in SHM. Indeed, we found a strong, inverse relationship between E-box motif fidelity and mutation frequency. Moreover, E2A was found to associate with the V region locale in two human B cell lines. Finally, analysis of human SHM datasets revealed that naturally occurring mutations in the 3’G flanking residues, which effectively ablate the E-box motif, were associated with a significantly increased rate of AGCT mutation.

**Discussion:**

Our results suggest an antagonistic relationship between mutation frequency and the binding of E-box factors like E2A at specific AGCT motif contexts and, therefore, highlight a new, suppressive mechanism regulating local SHM patterns in human V regions.

## Introduction

Somatic hypermutation (SHM) of immunoglobulin (IG) genes in B cells is essential for producing high-affinity antibodies against antigens on pathogens and vaccines (1). SHM occurs within germinal centers of secondary lymphoid tissue where iterative cycles of mutation and antigen-mediated affinity selection result in the clonal expansion of B cells expressing antibodies with higher affinity towards the target antigen (2). Point mutations are introduced into the variable (V) region of the IG heavy chain (*IGH*) and light chain genes by the enzyme, activation-induced deaminase (AID) (3, 4), which deaminates cytosine to uracil on single-stranded DNA (ssDNA) in a transcription-dependent manner (5–8). AID preferentially acts on WRCH hotspots (where W=A/T, R=A/G and H=A/C/T) (9–11). The U:G mismatch can result in a C>T transition mutation upon replication, while induction of error-prone repair mechanisms such as base excision repair can lead to C>G or C>A transversions (12, 13). Additionally, mismatch repair pathways generate mutations at A/T residues surrounding the U:G mismatch (12, 13). A striking and consistent feature of SHM profiles is the differential mutability of WRCH motifs wherein mutation frequencies of WRCH motifs vary substantially, not only between different motifs but also between multiple occurrences of identical motifs within a V region (14–18). This has led to the idea that the sequence context of these motifs plays a major role in determining their mutability (14–18). This idea has recently received important experimental support from a study which showed that the density of pyrimidine dimers (PyPy) in the 6 nt region upstream of AGCT motifs correlates with increased mutability, perhaps because PyPy richness confers flexibility to ssDNA that may facilitate AID targeting (19). Therefore, a major effort in the field is to further understand the mechanisms regulating differential mutability during SHM.

The recruitment of AID to V regions and other IG and non-IG targets has been linked to specific activating chromatin modifications (20–29) and transcriptional and co-transcriptional activities, notably, RNA polymerase II pausing (30–36), RNA exosome-mediated processing of RNA: DNA hybrids (37, 38) and convergent transcription (39). However, nascent transcriptional profiling of multiple V regions and hundreds of non-IG AID target loci at single-nucleotide resolution revealed no apparent correlation between mutation frequency of specific WRCH motifs and transcriptional strength or transcriptional features in its neighborhood (40). Thus, although transcriptional activities and chromatin marks are important for recruiting AID to its genomic targets, the observed differential mutability characteristic of SHM patterns cannot be explained solely by the transcriptional landscape (40). This finding further supports the notion that, following AID recruitment to V regions, the relative mutation frequency of WRCH motifs likely depends on the sequence neighborhood of each motif.

The major *cis*-regulatory elements regulating SHM are the IG enhancers which harbor binding sites for a plethora of transcription factors (TFs) (41–45). Amongst these, the E-box-binding TF, E2A, has been linked to SHM in multiple studies (42, 46–50). In experiments of enhancer-driven SHM of reporter substrates, elements with the E-box motif were found to have a particularly large impact on SHM, and among the TFs predicted to bind, loss of E2A was shown to cause a significant decrease in SHM (43). E2A, AID and other TFs were reported to form a complex that could associate with *IGH* (51, 52). It has also been shown that the presence of a E2A-binding motif enhances SHM in nearby regions (53) and may even facilitate AID recruitment (54).

To understand the mechanisms of differential mutability, our group recently developed DeepSHM, a convolutional neural network model trained to predict mutation frequencies of the central nucleotide in a 5-mer, 9-mer, 15-mer or 21-mer motif derived from human SHM data (18). The model achieved a high cross-validated Pearson correlation of r=0.81 with the experimental data (23). Moreover, model performance did not improve beyond a 15-mer context, that is, a 21-mer context did not significantly improve the predictions (18). In addition to the advantage brought forth by the expanded sequence context, compared to previous work which used shorter window sizes of 5–7 nucleotides (55), this approach also allows for use of interpretability techniques, which can be used to understand the model’s reasoning behind its predictions and, therefore, gain insights into potential biological mechanisms (18).

In this study, we extend the use of interpretability methods on DeepSHM to investigate the basis for positional differences in mutability of AGCT, one of the most frequently mutated WRCH motifs in human V regions. We report that conserved AGCTs near the 5’ end of V regions undergo significantly lower SHM than other AGCTs and that this suppression of mutability coincides with the presence of an E2A-binding E-box motif. We find that E2A is associated with V regions. The negative impact of E-box motifs was independent of the positive effect of PyPy richness. Ablation of this motif through naturally occurring mutations correlated with significantly increased mutation frequency. Thus, our study highlights a potential mechanism by which local sequence context negatively regulates mutability and contributes to the discrete SHM profiles of V regions.

## Materials and methods

### Sequence data

The 15-mer sequence dataset used to train DeepSHM was generated as described in our previous publication (18) and is available at https://gitlab.com/maccarthyslab/deepshm. Germline IGHV reference sequences from the international ImMunoGeneTics information system (IMGT) (56) were downloaded and split into k-mers using a sliding window approach. Mutation frequencies for the central nucleotide in each k-mer were calculated by comparing against a B cell receptor (BCR) sequencing (BCR-seq) dataset from marginal zone, memory, and plasma B cells from healthy volunteers, as described in our previous study (57). To study intrinsic SHM patterns and avoid confounding issues arising from clonal selection in germinal centers, we used only non-productive sequences (containing internal frameshifts or stop codons) and clonally independent sequences (one sequence per clone, as assigned by Change-O (58), which uses CDR3s to segment clones) (57).

For this study, only 15-mers containing an AGCT motif, with either the G or the C as the central nucleotide, were used with DeepSHM to predict mutation frequencies. The total 15-mer dataset was processed to extract those containing AGCTs using custom Python scripts. Our statistical analysis of synonymous mutations (those that do not change the protein sequence) ablating the CAGCTG motif was done with productive, clonally independent sequences. All statistical tests were performed using SciPy (59).

### DeepSHM

DeepSHM (https://gitlab.com/maccarthyslab/deepshm) is a deep learning model that uses a convolutional neural network architecture to predict mutation frequency or substitution rate of the central nucleotide in a k-mer of size 5, 9, 15 or 21. We used the 15-mer mutation frequency model, which takes a DNA sequence of 15 nucleotides as input and outputs a predicted mutation frequency value between 0 and 1. The 15-mer sequences were encoded into a 4 x 15 binary matrix, with rows corresponding to the 4 nucleotides and columns to the 15 positions along the k-mer. In each column, a 1 was placed in the appropriate row to denote the base identity for that position, while the remaining rows were 0s. This procedure, called one-hot encoding, is a common method for converting categorical data (A, G, C, T) into a machine-readable format (0s and 1s).

We downloaded the h5 file containing the model (model_15_mf.h5) and used it with Python to predict mutation frequencies for AGCT 15-mers in our dataset.

### Integrated gradients

Integrated gradients is an attribution method that measures the impact of individual inputs towards the output prediction of a deep learning model (60). It relies on a baseline value to compute a path integral of the model’s gradients with respect to its inputs, from the baseline to the input value. Since our input data is binary, we used a zero-matrix as our baseline with 50 steps taken from baseline to input. All 15 nucleotides in the 15-mer are considered as input features in the prediction of the mutation frequency of the central nucleotide, hence integrated gradients calculates a score for each base according to its impact on the output prediction.

The following GitHub repository was used to compute the integrated gradients scores for each of the DeepSHM predictions: https://github.com/hiranumn/IntegratedGradients. The repository was cloned and imported into the python script where the DeepSHM predictions were being run and used to compute integrated gradients scores for each input in each prediction. We generated sequence logo plots to visualize the frequency of nucleotides occurring at each position across 15-mers in each subregion using Logomaker (61), which is available at the following GitHub repository: https://github.com/jbkinney/logomaker.

### MOODS

MOODS (https://github.com/jhkorhonen/MOODS) is a position-weight matrix (PWM) matching algorithm that takes sequences and a counts matrix as inputs and outputs match scores for a segment of the sequences (62). The counts matrix is a 4×n matrix where the rows correspond to nucleotides (A, G, C, or T) and the columns correspond to positions along the TF binding motif, with the number of counts for each nucleotide in each position empirically obtained using SELEX and available on the JASPAR database (63). MOODS uses log-likelihood scoring to convert the counts matrix to a PWM, which it then compares against the sequence to generate a match score, only reporting scores at positions that exceed a *P* value cutoff of 0.001. We used MOODS to gauge the fidelity of our 15-mer sequences to binding motifs for the E-box TFs, E2A (https://jaspar2020.genereg.net/matrix/MA0522.2/) and TFAP4 (https://testjaspar.uio.no/matrix/MA1570.1/).

### ChIP-seq data

The E2A ChIP-seq data in Ramos cells was taken from a previous study (45) and is available at https://www.ncbi.nlm.nih.gov/bioproject/587064. The E2A ChIP-seq data in GM12878 cells was taken from ENCODE (64) and is available at https://www.encodeproject.org/experiments/ENCSR000BQT/. Both datasets were subject to the same analysis pipeline - a local alignment to hg38 of both case and control datasets using bowtie2 (65), sorting and indexing the resulting bam file using samtools (66). The callpeaks function of MACS2 (67) was run on the aligned bam files using the default q-value cutoff of 0.05 to call peaks. These peaks were then subject to motif enrichment analysis using the findMotifsGenome.pl function of the HOMER suite (68), using the hg38 genome and default size setting of 200 bp. The RPKM calculation was conducted using deeptools bamCoverage (69) with the bin size parameter set to 500 bp.

## Results

### DeepSHM recapitulates the observed positional differences in AGCT mutability

V regions can be structurally divided into antigen-binding complementarity-determining regions (CDR1–3) and intervening structural framework regions (FW1–3). AGCT is one of the most highly mutated WRCH motifs in V regions (70). The fact that AGCT is palindromic increases the probability that AID will deaminate cytosines on both the forward and the reverse strands (70). All human IGHV genes (at least the *01 IMGT alleles), except for three from the IGHV2 family, have one or more AGCT motifs near the 5’ end located in FW1 (57).

**Figure 1:**
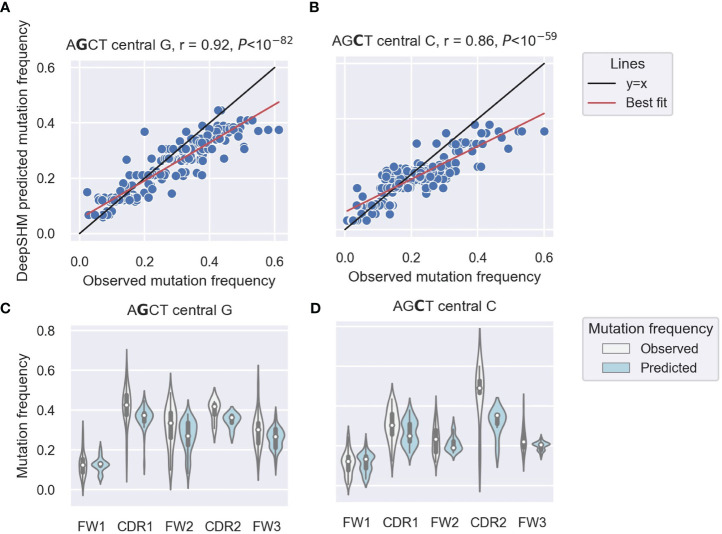
DeepSHM model performance on AGCT 15-mers. **(A, B)** Correlation scatter plots between observed and DeepSHM-predicted mutation frequencies. 15-mers centered on G (AGCT) **(A)** or C (AGCT) **(B)**. Each dot represents a 15-mer, the black line is the x=y diagonal and the red line indicates the best fit with intercept and coefficient computed using a linear regression. The r value is the Pearson correlation coefficient, and the *P* value is computed using a Wald test. **(C, D)** Violin plots showing the distributions of observed (white) and DeepSHM-predicted (blue) mutation frequencies for AGCT 15-mers within CDR and FW regions centered on G (AGCT) **(C)** and C (AGCT) **(D)**. The white dots represent the median, the black boxes show the interquartile range, and the whiskers encapsulate points that fall between 1.5 times the inter-quartile range.

Our previous analysis of IGHV3–23*01 non-productive sequences showed higher mutability of AGCTs in the CDRs and a particularly low mutability of the 5’ AGCT in FW1 (14). To examine this differential mutability of AGCT motifs across all IGHV genes, we used DeepSHM to predict mutation frequencies of the central nucleotides in the AGCT 15-mers in our dataset and compared the results with the observed data using a correlation analysis. We achieved a Pearson correlation of r=0.92 for those with a central G site (**Figure 1A**) and r=0.86 for those with a central C (**Figure 1B**). These high correlations suggested that the neural network had identified sequence features that distinguish low from high mutation frequencies for AGCT sites. To confirm that the positional differences in AGCT mutability previously observed for IGHV3–23*01 applied to other IGHV alleles, we examined our dataset of 16,870 15-mers across 65 IGHV alleles and their associated mutation frequencies (57). We separated the AGCT 15-mers in our dataset by IMGT subregion and plotted the observed and predicted mutation frequencies for those with central Cs (**Figure 1C**) and central Gs (**Figure 1D**). We observed a statistically significant difference in mutation frequency between the AGCT motifs in FW1 and all other subregions for both observed and predicted datasets centered on the G (t-test, *P*<10^–30^) (**Figure 1C**) and C (t-test, *P*<10^–20^) (**Figure 1D**).

We conclude that that AGCTs in FW1 are significantly less mutated than those in other V subregions and that DeepSHM can recapitulate these observed positional differences in AGCT mutability.

### Integrated gradients reveals a sequence context associated with decreased mutability of FW1 AGCT motifs

To interrogate the specific sequence features associated with high or low mutation frequency predictions, we used an interpretability method, integrated gradients (22). Integrated gradients analyses involve computing the derivative of the output (mutation frequency prediction) with respect to the input (15-mer sequence) to ascribe importance to input features based on their impact on the output prediction. Specifically, a higher integrated gradients score would imply that a small change in input had a more positive contribution towards the output prediction. Conversely, lower integrated gradients scores indicate that changes in input features contributed negatively to the predicted output.

**Figure 2:**
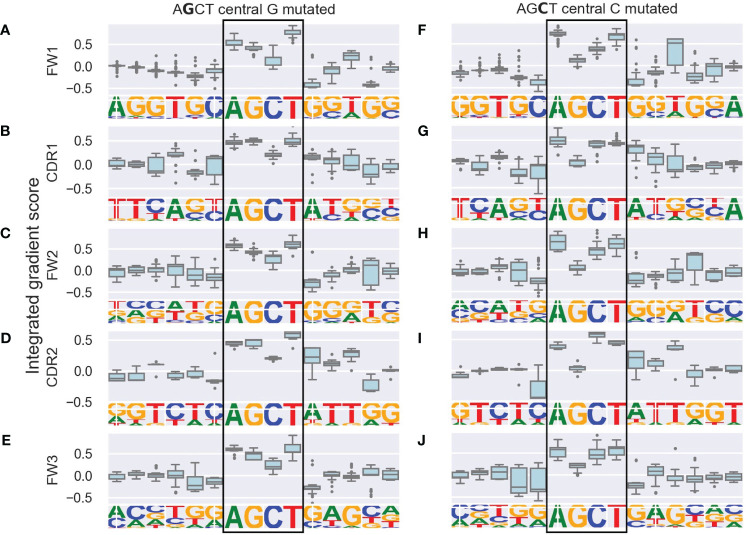
Integrated gradients scores for each nucleotide in AGCT 15-mers across V subregions shown as boxplots. The left column consists of sequences with a central G **(A–E)** and the right column consists of sequences with a central C **(F–J)**. Rows correspond to the indicated V subregion and the sequence logo below each boxplot corresponds to the nucleotide frequency at each position. The boxes represent the inter-quartile region of the distribution of integrated gradient scores for each nucleotide, with the black line through the box showing the median score and the whiskers representing 1.5 times the inter-quartile range. Outlier points are shows as dots. Note that nucleotides in the central AGCT hotspot (boxed) tend to have the largest scores in the 15-mer and that the 5’ and 3’ flanking nucleotides for the FW1 AGCT **(F)** have the lowest scores in the 15-mer.

We generated integrated gradients scores for each nucleotide within the AGCT 15-mers for its prediction of (i.e. contribution towards) the mutation frequency of the central G or C within the hotspot. We plotted the range of scores for each position as boxplots which were further categorized based on the location of the 15-mers within FW and CDR subregions (**Figures 2A–J**).

As a positive control, we observed that nucleotides within the AGCT hotspot across all central G 15-mers (**Figures 2A–E**) and 85% of central C 15-mers (**Figures 2F–J**) had a positive integrated gradients score, meaning that the presence of these nucleotides increased the mutability prediction of the central G or C. We then examined the integrated gradients scores for FW1 AGCTs (**Figures 2A, F**) as they are significantly less mutated than AGCTs in other V sub-regions (**Figure 1C**). We found that 78% of the lowly mutating FW1 AGCTs were flanked by a 5’-C and 3’-G nucleotide, both of which have large negative integrated gradient scores (**Figure 2F**). This suggests that an extended CAGCTG motif context decreases the mutability prediction for the central G and C nucleotides within these lowly mutating FW1 AGCTs, implying that CAGCTG motifs may be less frequently targeted by AID.

**Figure 3:**
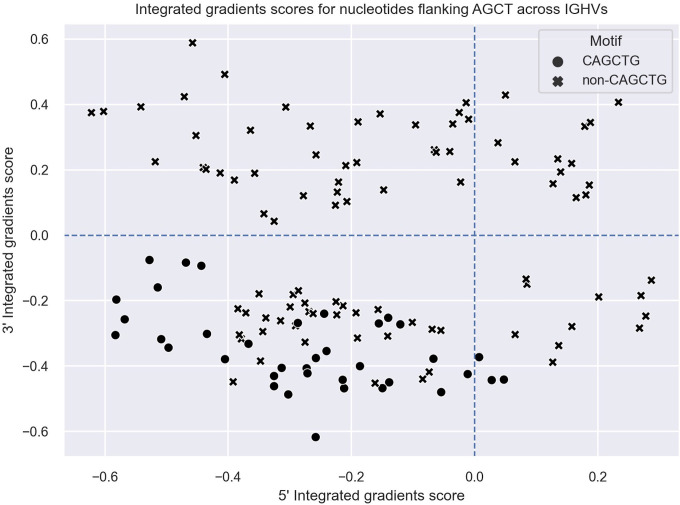
Integrated gradients scores for the 5’ and 3’ flanking nucleotides of AGCT motifs across all human V regions shown as a scatter plot. Each dot/cross corresponds to a 15-mer. 15-mers in which the central AGCTs are flanked by 5’-C and 3’-G (CAGCTG motifs) are indicated with a cross (x).

**Figure 4:**
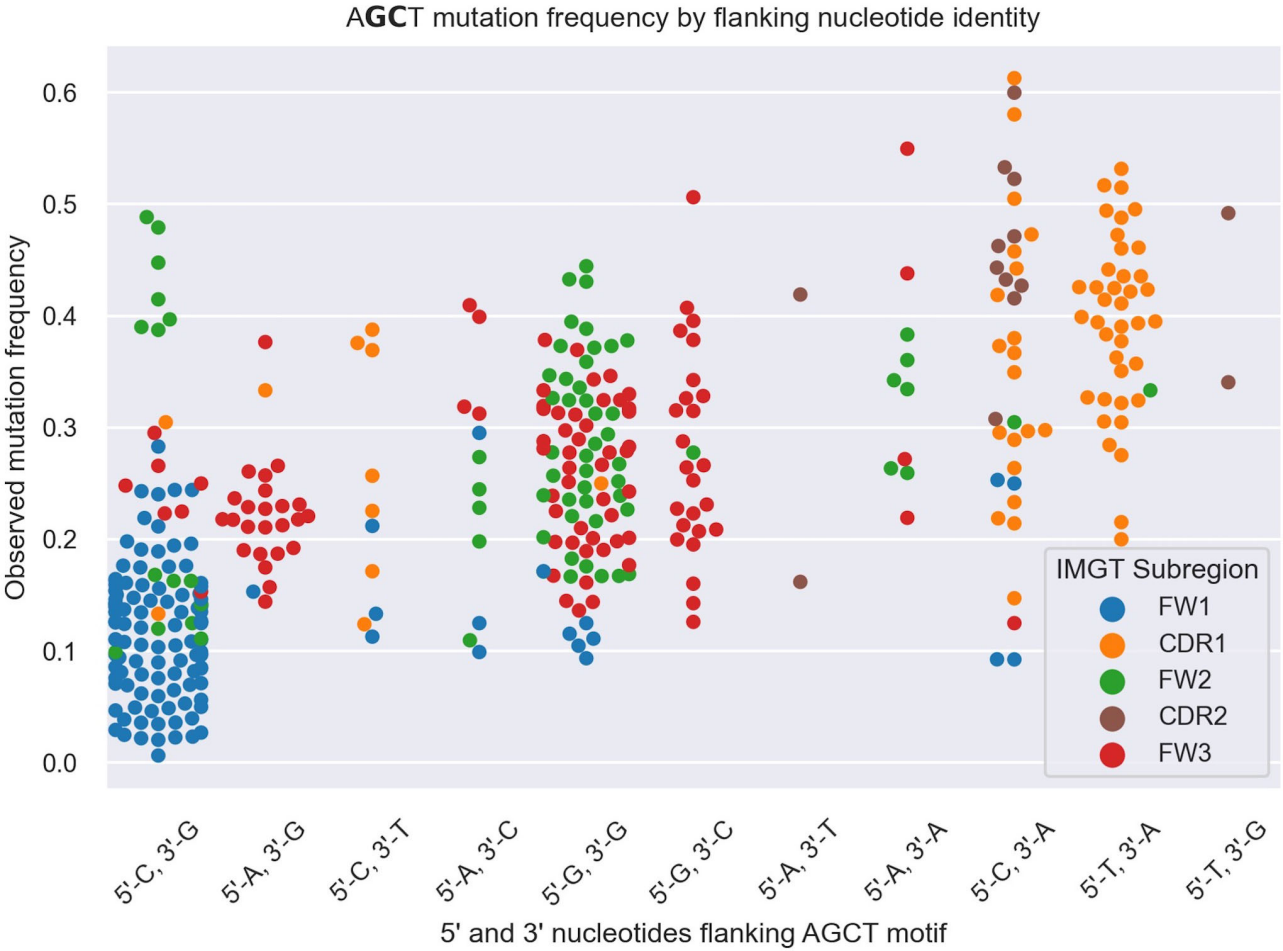
Swarm plot depicting mutation frequencies for the central G and C residues within AGCT 15-mers categorized based on the identity of the 5’ and 3’ nucleotides flanking AGCT. The color coding highlights the location of the 15-mer in CDRs or FWs. Each AGCT is represented by two dots - one for the central C and one for the central G. AGCT motifs flanked by 5’-C and 3’-G, corresponding to the CAGCTG motif (first category on the left), has a significantly lower mutation frequency (*P*<10^–30^) than any other pair as computed by a Mann-Whitney U Test.

To explore this idea further, we directly compared integrated gradients scores of the 5’ and 3’ nucleotides flanking AGCTs across all 15-mers. We found that for CAGCTG-containing 15-mers, the integrated gradients scores were almost always negative for the 5’-C (98%) and consistently negative for the 3’-G (100%), supporting the notion that the CAGCTG context has a predominantly negative influence on mutagenesis of AGCT (**Figure 3**). Additionally, of all AGCT flanking nucleotide combinations, the 5’-C and 3’-G combinations were overwhelmingly within the FW1 region and were significantly less mutated than AGCTs with other flanking nucleotide combinations (Mann-Whitney test, *P*<10^–30^) (**Figure 4**). We note that although most of the FW1 AGCT motifs were flanked by 5’-C and 3’-G nucleotides, even those flanked by other nucleotide combinations tended to have lower mutation frequencies (blue dots in **Figure 4**). This suggests that the position of the AGCT within the V region may also have some influence on its mutability. In addition, a fraction of CAGCTG motifs in FW2 undergo higher mutation frequency than those in FW1 (**Figure 4**), suggesting the presence of additional mechanisms, possibly involving differences in the larger sequence context of these motifs, that influence differential mutability, which we address in the following section.

We conclude that the weakly mutated AGCTs in FW1 are predominantly flanked by 5’C and 3’G nucleotides, implying that the CAGCTG sequence context correlates with reduced SHM of AGCT motifs.

### CAGCTG is an E-box binding motif and E2A associates with V regions

CAGCTG corresponds to the CANNTG E-box binding motif of the basic helix-loop-helix TF family, which includes E2A (50). To predict binding probabilities of E2A to AGCT 15-mers, we used MOODS, a TF-binding prediction package which utilizes counts matrices obtained from empirical SELEX data, wherein higher MOODS scores reflect a stronger sequence match to a particular TF binding motif (62).

**Figure 5:**
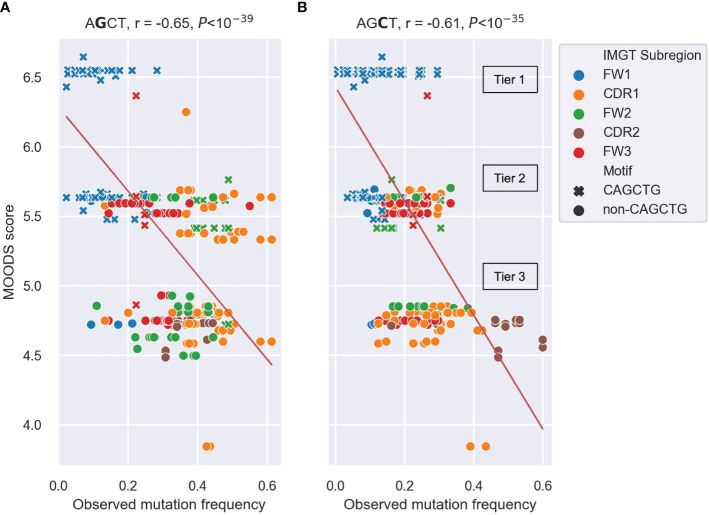
Scatter plots depicting the correlation between observed mutation frequencies and E2A MOODS scores for AGCT 15-mers. **(A, B)** analysis of 15mers centered at the central G **(A)** or central C **(B)**. Each point represents a 15-mer and is colored by IMGT subregion, with CAGCTG 15-mers indicated with a cross (x). The red lines indicate the best fit with intercept and coefficient computed using a linear regression. The r value is the Pearson correlation coefficient, and the *P* value is computed using a Wald test. The three tiers (Tier 1–3) that the MOODs scores fall into are labeled.

We found significant negative Pearson correlations of r=-0.64 and r=-0.65 between the MOODS binding scores for E2A motifs and the mutation frequencies of the central Gs (**Figure 5A**) and central Cs (**Figure 5B**) in the AGCT sites, respectively. Similar analysis for TFAP4, another E-box TF commonly expressed in B cells, showed weaker correlations (**Supplementary Figure 1**). The predicted MOODS scores for the AGCT 15-mers fell roughly into three discrete tiers. Tier 1, having the highest MOODS scores but generally lower mutation frequencies, and consisting almost entirely of CAGCTG-containing 15-mers (**Figures 5A, B**). Tier 2, having intermediate MOODS scores with a wide range of mutation frequencies. Importantly, although this tier consists of a mixture of CAGCTG and non-CAGCTG 15-mers, the former showed a tendency to be less mutated than the latter (**Figures 5A, B**). Interestingly, the FW2 CAGCTG 15-mers observed to be highly mutating in **Figure 4** fall into this tier and contain central Gs (green crosses in **Figure 5A**). This indicates lower fidelity to the E2A motif than the lowly mutating FW1 CAGCTGs and may explain, in part, the higher mutation frequency due to diminished E2A binding. Tier 3, which harbored the lowest MOODS scores and generally higher mutation frequencies, consisted mostly of non-CAGCTG 15-mers (**Figures 5A, B**). These results suggest that the binding probability of E2A to an AGCT-centered 15-mer negatively correlates with mutation frequency of that AGCT.

**Figure 6:**
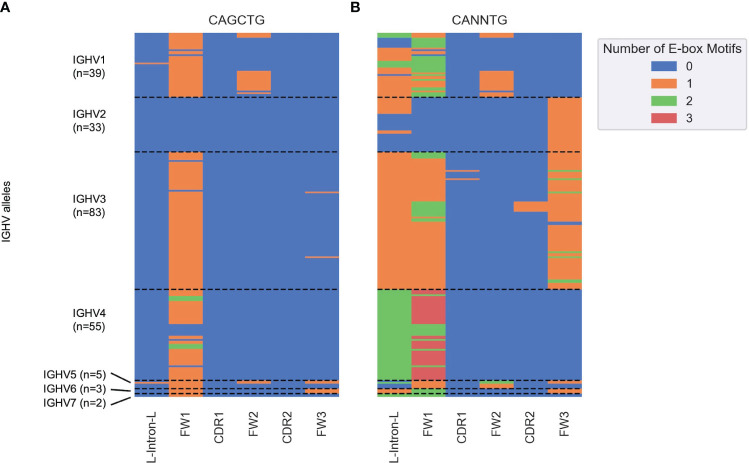
Heatmap depicting the number of **(A)** CAGCTG and **(B)** CANNTG E-box motifs in human germline IGHV genes (y axis) classified into subregions (x axis) based on the IMGT nomenclature. Each cell corresponds to a distinct IGHV sub-region and is colored by the number of E-box motifs (between 0 and 3) in that sub-region as shown in the key on the right. Each row corresponds to a unique IGHV allele. The dashed horizontal lines represent boundaries between the seven IGHV families (IGHV1–7).

Next, we determined the distribution of all potential E2A sites across all human IGHV genes. We segmented germline sequences for the 220 alleles obtained from the IMGT database into six subregions and counted the number of occurrences of CAGCTG (**Figure 6A**) and the more general CANNTG (**Figure 6B**) E-box motifs. CAGCTG motifs were mostly distributed in the FW1 region, with the IGHV2 family notably lacking them (**Figure 6A**). The IGHV4 family has the highest density of CANNTG motifs while most of IGHV2 family have E-box motifs in the FW3 region (**Figure 6B**). Overall, each of the 220 alleles had at least one E-box motif, with most of them in FW1 and/or the leader-intron-leader (L-intron-L) sequence which immediately precedes FW1 (**Figure 6B**).

**Figure 7:**
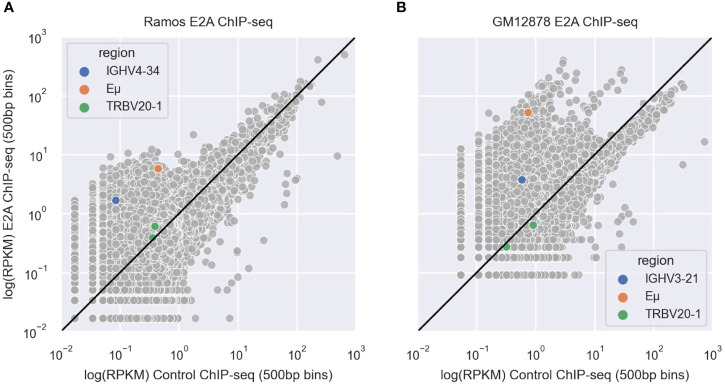
**(A, B)** Scatter plots depicting correlations between IgG control and E2A ChIP-seq shown as reads per kilobase million (RPKM) values in 500 bp genomic bins for Ramos **(A)** and GM12878 **(B)** cells. Bins containing the rearranged IGHV, Eμ enhancer and TRBV20–1 are highlighted in blue, orange and green, respectively. The black line represents the y=x diagonal.

To determine whether E2A associates with IGHV regions, we analyzed E2A ChIP-seq datasets derived from Ramos (45) and GM12878 (64) B cell lines. After aligning these data to the hg38 reference genome using bowtie2 (65), we used MACS2 (67) to call peaks and then conducted a motif enrichment analysis using HOMER (68). We saw that for both Ramos (**Supplementary Table 1**) and GM12878 (**Supplementary Table 2**) cell lines, the CAGCTG motif corresponding to the E2A TF binding motif was highly enriched and among the top two most significant results. We calculated the reads per kilobase million (RPKM) values of 500bp bins in both the E2A ChIP-seq and the IgG control ChIP-seq alignments and plotted their correlations. In both Ramos (**Figure 7A**) and GM12878 (**Figure 7B**) cells, the bins containing the rearranged V region (IGHV4–34 in Ramos (71) and IGHV3–21 in GM12878) (72)) were enriched for E2A binding, as was the bin containing the *IGH* Eμ enhancer, which serves as a positive control for E2A binding (43). However, bins containing a negative control region, TRBV20–1, a commonly used T-cell receptor V gene (73), showed no enrichment for E2A binding in either cell line (**Figures 7A, B**). Thus, E2A can directly associate with V regions.

Altogether, these results suggest that E2A association with CAGCTG motifs in FW1 suppress AID targeting to these AGCTs, thereby providing a plausible explanation for the strong negative correlation between this motif context and mutation frequency.

### E-box and PyPy dimers contribute independently to AGCT mutability

We additionally sought to compare the role of E2A binding with another sequence-level determinant of AGCT mutability proposed in a recent study (19), namely, the presence of PyPy dimers. In this study, a higher frequency of PyPy dimers in the 6 nucleotides 5’ to the AGCT was associated with increased mutation frequency of the central C residue (19). Therefore, we counted the number of PyPy dimers in the 6 nt region immediately upstream of AGCT motifs in our k-mer dataset and fit a linear regression model predicting mutation frequency, including an indicator variable for the presence of the E-box motif. This binary E-box indicator variable had a Pearson correlation of r=-0.61 with the mutation frequency, while the integer PyPy count had a correlation of 0.51 (**Table 1**). Importantly, therefore, both variables individually correlate with mutation frequency in directions consistent with our expectations, that is, positive for PyPy counts, which increases mutation frequency, and negative for the presence of an E-box, which reduces mutation frequency.

**Table 1:** Pearson r and R^2^ for correlations and model performance against mutation frequency.

Variable	Pearson r	R^2^
PyPy Counts	0.51	0.25
E-box indicator	-0.61	0.39
PyPy + E-box	N/A	0.45

Given this trend, we expected a linear model with both variables to have a higher performance than a model with either individual variable, as measured by R^2^, which directly reflects the proportion of variance in the output variable (mutation frequency) explained by the input variables (E-box motif, PyPy richness, or both). The combined regression model achieved an R^2^ of 0.45 meaning that 45% of the variance in mutation frequency is explained by the presence of E-box and PyPy motifs (**Table 1**). The regression model with only the PyPy counts variable achieved an R^2^ of 0.25 while the model with only the E-box indicator variable achieved a higher, and closer to the combined, R^2^ value of 0.39 (**Table 1**). Of note, the coefficients generated by the model had signs appropriate to the direction of correlation with mutation frequency, that is, positive for PyPy counts and negative for the E-box indicator (**Table 1**).

These results lead to the conclusion that both mechanisms, decreasing mutation frequency of FW1 AGCTs, plausibly through E2A binding, and increasing mutation frequency of AGCTs through increased AID binding to flexible PyPy-rich DNA can contribute independently to the observed mutability. Importantly, however, the R^2^ values observed from these analyses also imply that additional mechanisms are necessary to fully explain AGCT mutability.

### Ablation of the CAGCTG motif is associated with a significant increase in mutation frequency

To better understand the relationship between the E-box motif and the mutability of the central nucleotides, we examined mutations of the CAGCTG hotspot in FW1. We hypothesized that if this motif context negatively contributes to SHM, then naturally occurring mutations that ablate this context would be expected to increase mutation frequency of the AGCT within it.

Due to the paucity of non-productive sequences in our dataset, we examined synonymous mutations (i.e. those that do not cause changes in protein sequence) in productive BCR sequences to preclude any effect of affinity selection. Specifically, we focused on the G residues at positions 3 (G_3_) and 6 (G_6_) of CAGCTG. Importantly, the FW1 CAGCTG motif occurs at position 7–12 of the V segment and is always in frame, such that mutations at G_3_ and G_6_ are in the third position of their respective codons. Thus, G_3_>A_3_ mutations are synonymous since CAG and CAA are degenerate codons for glutamine. Similarly, G_6_>H_6_ mutations (where H = A/C/T) are also synonymous since CTG, CTA, CTC and CTT are degenerate codons for leucine. Importantly, G_6_>H_6_ mutations (CAGCTH) would ablate the E-box motif. Thus, we compared mutation frequency at the central AGCT in clonal groups having an unmutated CAGCTG in FW1 or a CAGCTH in the same position.

**Figure 8:**
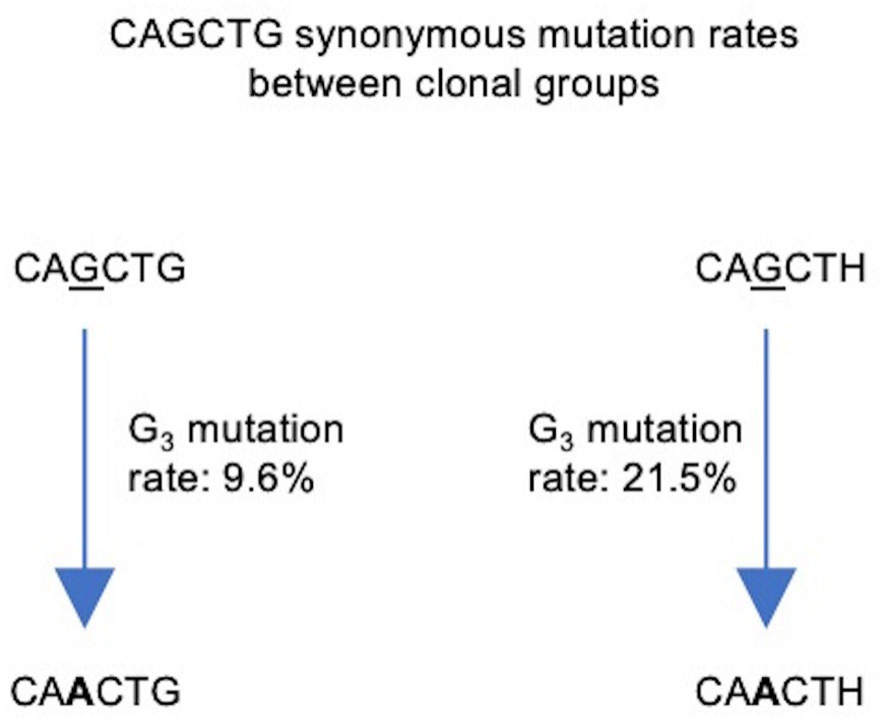
Flowchart depicting synonymous mutations of the Gs at site 3 (G_3_) of the CAGCTG E-box motif in FW1. Calculated mutation frequencies of G_3_ sites before and after mutation of G_6_ are indicated beside the arrows. Calculations were conducted using productive, clonally independent sequences across clones.

To prevent double counting of mutations occurring during clonal expansion, we selected a sequence at random from each clonal group (57). Our sequence data consisted of 642,367 clonal groups, of which 504,333 had a sequence identity in position 7–12 of the V segment, corresponding to one of the four motifs of interest: unmutated CAGCTG (G_3_/G_6_), single mutant CAACTG (A_3_/G_6_), single mutant CAGCTH (G_3_/H_6_) and double mutant CAACTH (A_3_/H_6_). We counted the number of occurrences of each (**Table 2**) and used these numbers to calculate mutation frequencies for sites 3 and 6 (**Supplementary Table 3**). We observed that the mutation frequency of G_3_ increases from 9.6% when G_6_ is unmutated to 21.5% when G_6_ is mutated, a highly significant difference (Fisher test, *P*<10^–16^) (**Figure 8**). Thus, the presence of an intact CAGCTG motif is strongly associated with lower mutation of the AGCT within it.

**Table 2:** Counts of synonymous mutations at sites 3 and 6 of the CAGCTG motif across clones.

Site 6/Site 3	Unmutated (G_3_)	Mutated (A_3_)
Unmutated (G_6_)	426962	45537
Mutated (H_6_)	24988	6846

Altogether, our results support the notion that FW1 AGCT motifs occurring in the context of the E-box binding motif, CAGCTG, lead to dampened SHM in these locales.

## Discussion

In this study, we use interpretable deep learning to provide evidence for the role of DNA sequence context in negatively modulating SHM at AGCT motifs located at the 5’ end of most human IGHV genes, except those of the VH2 family. Our work suggests that the occurrence of this AGCT in the context of a CAGCTG E-box motif correlates strongly with reduced SHM. Together with the fact that E2A can associate with VH4–34 and VH3–21, we propose that SHM may be dampened at these motifs, at least in part, by the association of E-box-binding TFs. The decrease in AID mediated mutations could occur through a variety of mechanisms including changes in transcription elongation or pausing, or a decrease in the recruitment of AID or its associated cofactors. In effect, this would constitute a new, suppressive mechanism contributing to the differential mutability of AGCT motifs in specific contexts. Our work, therefore, provides a conceptual framework to guide further studies aimed at identifying similar mechanisms regulating local SHM probabilities at other WRCH motifs, including other AGCT motif contexts, perhaps involving different TFs or combinations thereof.

How might E2A binding to CAGCTG suppress SHM? E2A binds ssDNA *in vitro* and has a higher affinity for CAGCTG than for the canonical dsDNA binding site, CAGGTG (74). Additionally, mutations in the middle nucleotides of the CANNTG motif reduced E2A binding to ssDNA substantially, but not to dsDNA (74). These results, along with our analyses, suggest a competitive binding model for the significantly weaker mutability of the FW1 AGCT motifs wherein E2A binding to ssDNA may prevent AID from accessing exposed CAGCTG motifs. Since the CAGCTG E-box TF binding motif is palindromic, E2A could potentially access both strands, for instance, under conditions of transcription-induced negative supercoiling where both template and non-template strands can acquire transient ssDNA states (75). If so, E2A could restrict AID from accessing CAGCTG-containing ssDNA on either strand. Since the processing of SHM-induced mismatches in the V region can result in DNA double-strand breaks (76), we expect that such a mechanism would also impact on the formation of these lesions.

Collectively, these findings raise two hypotheses that merit further investigation. Firstly, other E-box-binding TFs expressed in B cells may associate with CAGCTG in FW1 and contribute to suppressing SHM. Secondly, AID accessibility at other WRCH motifs may be subject to similar negative regulation mediated by the competitive binding of different TFs. Such analyses are also necessary at non-IG SHM target loci implicated in B lymphomagenesis, such as *MYC* and *BCL6*, to ask whether similar mechanisms regulate differential mutability during off-target SHM.

Our analysis of PyPy richness revealed a positive correlation of this feature with AGCT mutability, in agreement with the *in vitro* findings of Wang et al. (19). Our results also suggest that PyPy-richness and E-box motifs can work independently in determining the mutability of AGCT motifs. Thus, we conclude that SHM enhancement via increased ssDNA flexibility conferred by PyPy motifs and SHM suppression via E2A binding at E-box motifs constitute two distinct mechanisms to achieve differential mutability of AGCT motifs. Importantly, however, it is evident from our data that these features, either singly or in combination, cannot fully explain AGCT mutability, implying that additional as-yet-unknown mechanisms exist that contribute to differential mutability, such as the influence of position, as in the case of some lowly mutating FW1 AGCTs that do not lie in a CAGCTG context (**Figure 4**).

In conclusion, our study reveals the complexity underlying local AID targeting and argues that the eventual discrete SHM profiles result from multiple mechanisms that either strengthen or dampen SHM. As exemplified by our study, deep learning tools will be an important resource for mining mutational datasets to gain further insights into these mechanisms.

## Data availability statement

The original contributions presented in the study are included in the article/**Supplementary Material**. All code use in this study is available at https://github.com/abhikt/e2a_paper. Further inquiries can be directed to the corresponding author.

## Author contributions

AT: Writing – original draft, Writing – review & editing, Data curation, Formal analysis, Investigation, Methodology, Validation, Visualization. TM: Writing – original draft, Conceptualization, Funding acquisition, Project administration, Resources, Supervision. RP: Supervision, Writing – original draft, Writing – review & editing.
