# Supplementary figures and images for: Interpretable deep learning reveals the role of an E-box motif in suppressing somatic hypermutation of AGCT motifs within human immunoglobulin variable regions
Source: Front Immunol. 2024 May 28;15:1407470. doi: 10.3389/fimmu.2024.1407470 (PMC11165027; doi:10.3389/fimmu.2024.1407470)

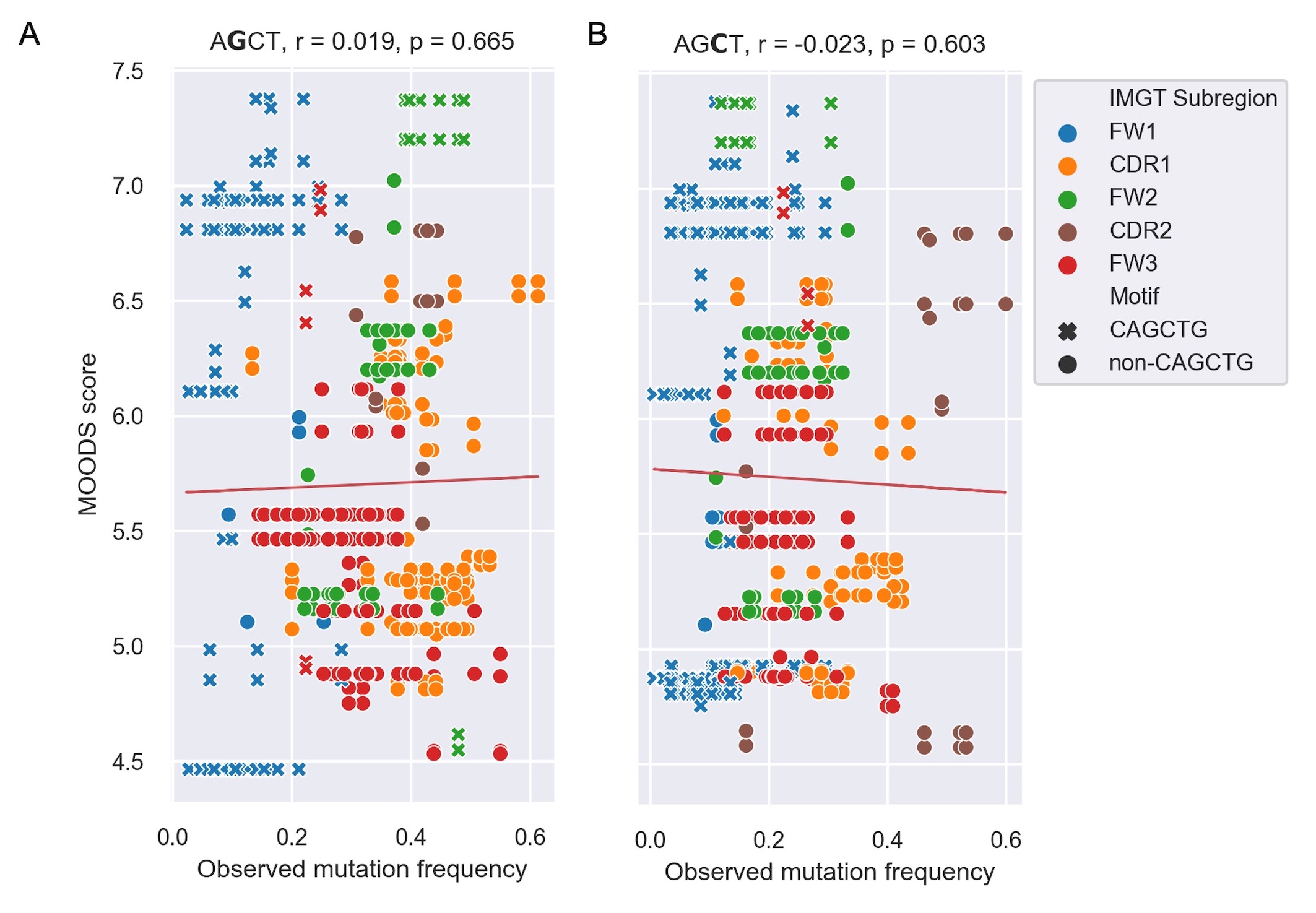

Supplement: Supplementary file 1 [file Image_1.jpeg]
