# Supplementary material for: Interpretable deep learning reveals the role of an E-box motif in suppressing somatic hypermutation of AGCT motifs within human immunoglobulin variable regions
Source: Front Immunol. 2024 May 28;15:1407470. doi: 10.3389/fimmu.2024.1407470 (PMC11165027; doi:10.3389/fimmu.2024.1407470)
Supplement: Supplementary file 2 [file Table_1.docx]

Supplementary Material

Interpretable deep learning reveals the role of an E-box motif in suppressing somatic hypermutation of AGCT motifs within human immunoglobulin variable regions

Abhik Tambe^1^, Thomas MacCarthy^2^, and Rushad Pavri^3,4*^

^1^Department of Biochemistry and Cell Biology, Stony Brook University, Stony Brook, NY, United States

^2^Department of Applied Mathematics and Statistics, Stony Brook University, Stony Brook, NY, United States

^3^Research Institute of Molecular Pathology (IMP), Campus-Vienna-Biocenter 1, 1030 Vienna, Austria

^4^Peter Gorer Department of Immunobiology, School of Immunology & Microbial Sciences, King’s College London, London SE1 9RT, United Kingdom

*Correspondence:
Rushad Pavri
rushad.pavri@kcl.ac.uk

**Supplemental figure legend**

**Figure S1**: Scatter plots depicting the correlation between observed mutation frequencies and TFAP4 MOODS scores for AGCT 15-mers. **(A-B)** analysis of 15mers centered at the central G (**A**) or central C (**B**). Each point represents a 15-mer and is colored by IMGT subregion, with CAGCTG 15-mers indicated with a cross (x). The red lines indicate the best fit with intercept and coefficient computed using a linear regression. The r value is the Pearson correlation coefficient, and the *P* value is computed using a Wald test. The three tiers (Tier 1-3) that the MOODs scores fall into are labeled.

**Table S1**: HOMER motif enrichment results for Ramos E2A ChIP-seq data

| Motif Name | Consensus | P-value | % Target Sequences with Motif | % Background Sequences with Motif |
| --- | --- | --- | --- | --- |
| E2A(bHLH)/proBcell-E2A-ChIP-Seq(GSE21978)/Homer | DNRCAGCTGY | 10^-779^ | 69.23% | 25.70% |
| HEB(bHLH)/mES-Heb-ChIP-Seq(GSE53233)/Homer | VCAGCTGBNN | 10^-736^ | 73.77% | 30.70% |
| Ascl2(bHLH)/ESC-Ascl2-ChIP-Seq(GSE97712)/Homer | SSRGCAGCTGCH | 10^-694^ | 58.46% | 19.41% |
| E2A(bHLH),near_PU.1/Bcell-PU.1-ChIP-Seq(GSE21512)/Homer | NVCACCTGBN | 10^-652^ | 64.18% | 24.69% |
| Ptf1a(bHLH)/Panc1-Ptf1a-ChIP-Seq(GSE47459)/Homer | ACAGCTGTTN | 10^-624^ | 78.17% | 38.22% |

**Table S2**: HOMER motif enrichment results for GM12878 E2A ChIP-seq data

| Motif Name | Consensus | P-value | % of Target Sequences with Motif | % of Background Sequences with Motif |
| --- | --- | --- | --- | --- |
| Ascl1(bHLH)/NeuralTubes-Ascl1-ChIP-Seq(GSE55840)/Homer | NNVVCAGCTGBN | 10^-2117^ | 39.09% | 20.14% |
| E2A(bHLH)/proBcell-E2A-ChIP-Seq(GSE21978)/Homer | DNRCAGCTGY | 10^-1822^ | 41.33% | 23.16% |
| HEB(bHLH)/mES-Heb-ChIP-Seq(GSE53233)/Homer | VCAGCTGBNN | 10^-1810^ | 47.42% | 28.43% |
| Ptf1a(bHLH)/Panc1-Ptf1a-ChIP-Seq(GSE47459)/Homer | ACAGCTGTTN | 10^-1734^ | 54.73% | 35.44% |
| Tcf12(bHLH)/GM12878-Tcf12-ChIP-Seq(GSE32465)/Homer | VCAGCTGYTG | 10^-1620^ | 26.03% | 12.08% |

**Conditional probability calculation**

Conditional probabilities of mutation of G_3_ and G_6_ in the CAGCTG motif were calculated from the counts data in **Table 2** using the following equation:

$$P\left( A | B \right)= \frac{P(A\cap B)}{P(B)}$$

**Table S3**: Conditional probabilities of mutations at sites 3 and 6 given base identity of the other site.

| Case | Condition | Conditional Probability |
| --- | --- | --- |
| A_3_ | G_6_ | 9.6% |
| A_3_ | H_6_ | 5.5% |
| H_6_ | G_3_ | 13% |
| H_6_ | A_3_ | 21.5% |
